# Adrenal Mass and Extensive Brown Fat Activation in a Young Female With Urinary Frequency and Hypertension

**DOI:** 10.1016/j.aed.2025.07.007

**Published:** 2025-07-28

**Authors:** Mary Shahinyan, Run Yu

**Affiliations:** 1 Freelance Researcher; 2 Division of Endocrinology, UCLA David Geffen School of Medicine, Los Angeles, California

## Case Presentation

**Fig. 1:**
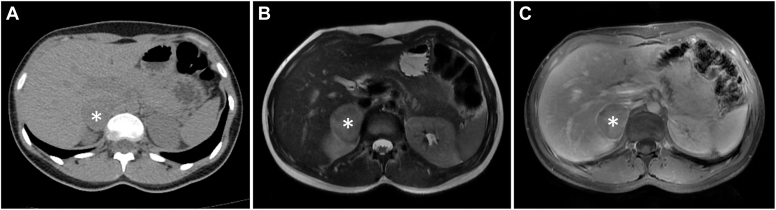

**Fig. 2:**
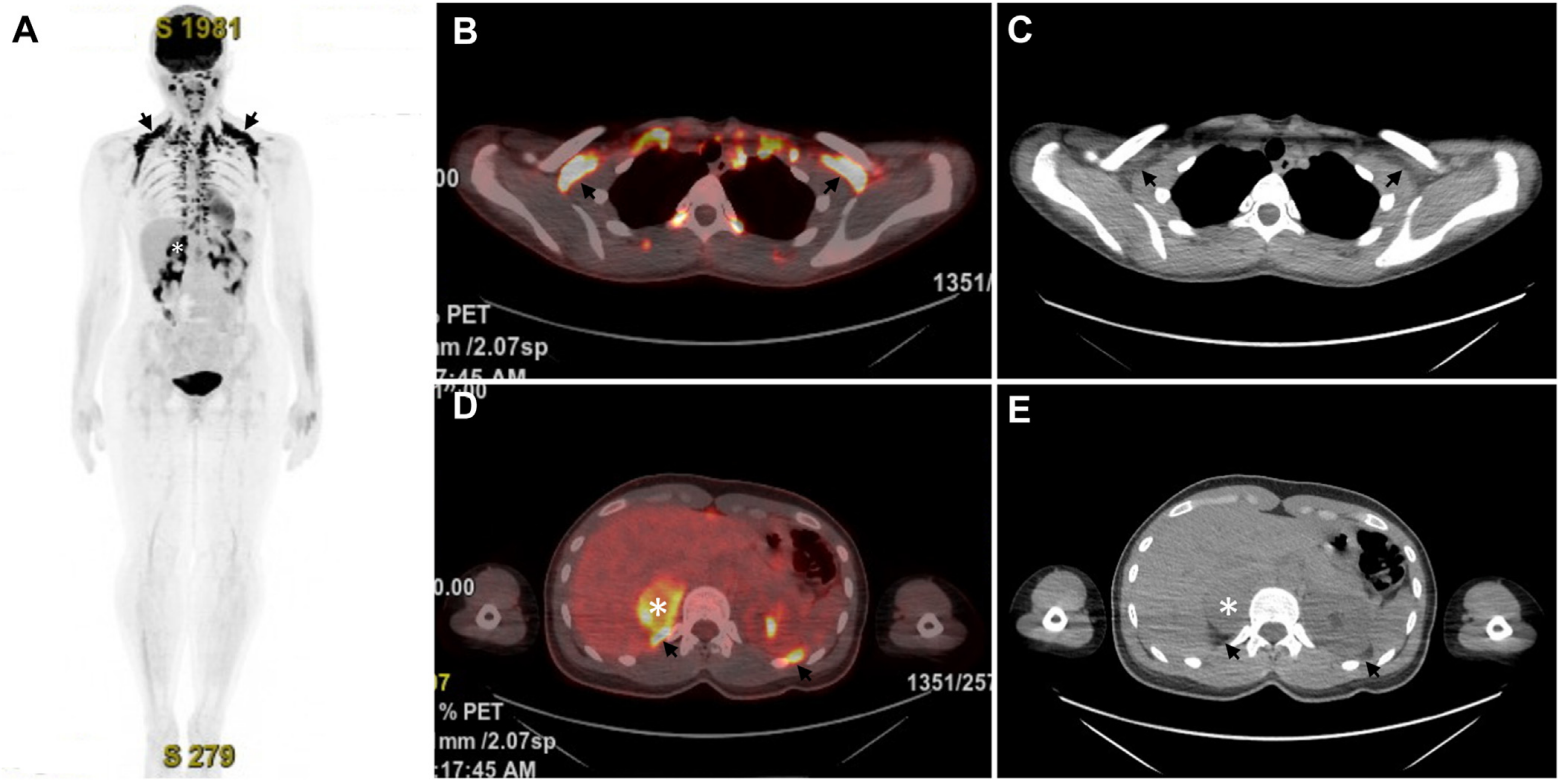

A 24-year-old woman was evaluated at the endocrine clinic for adrenal mass. Approximately 15 months before presentation, she had started experiencing nightly frequent urination with urinary urgency. Over the ensuing months, her symptom continued to worsen, and she was noted to have hypertension and started on amlodipine. Renal-bladder ultrasound for nocturia performed 6 months before presentation showed normal kidneys and bladder but incidentally identified a 3.5-cm hepatic lesion. Computed tomography (CT) of the abdomen was initially reported as normal; however, in retrospect, a right adrenal mass was clearly present and exhibited precontrast Hounsfield units of 30 to 40 (Fig. 1 *A*, asterisk). The patient’s urinary frequency worsened. A few weeks before presentation, magnetic resonance imaging (MRI) angiogram revealed a T2-bright (Fig. 1 *B*), heterogeneously enhancing (Fig. 1 *C*), 5.2-cm right adrenal mass. Fluorodeoxyglucose (FDG) positron emission tomography/CT demonstrated intense uptake in the adrenal lesion (Fig. 2, asterisk) and extensive areas of multifocal FDG-avid foci in supraclavicular, mediastinal, paravertebral, and perinephric fat (Fig. 2, arrows). No hypermetabolic lymph nodes or distant metastases were seen. Review of systems was positive for palpitations, weakness, headache, hot flashes with profuse sweating, and panic attacks, which had appeared 12 months after the urinary symptoms started. Her past medical history only included hypercholesterolemia. No family members had a history of endocrine tumors. Physical examination was only notable for hypertension.

## What is the diagnosis?

### Answer

Right adrenal pheochromocytoma with extensive brown fat activation. In this young patient with hypertension, an incidental adrenal mass with imaging features compatible with pheochromocytoma (precontrast Hounsfield units of >10 on CT, high signal on MRI T2 imaging, and enhancement on CT or MRI) raised high suspicion of this tumor.^1^ The intensive FDG uptake in the fat tissue foci typical for brown fat distribution was consistent with extensive brown fat activation, which can be seen in up to 20% of patients with pheochromocytoma, especially in younger ones such as this patient, due to high levels of circulating and local catecholamines released by pheochromocytoma.^2^ Catecholamines bind to β-adrenergic receptors on brown fat cells and increase production of uncoupling protein-1, drive up glucose uptake, and lead to extensive FDG uptake.^2^ Her plasma free normetanephrine level was 0.75 nmol/L (<0.49), and the free metanephrine level was 21.9 nmol/L (<0.89). Following α-blockade and subsequent β-blockade, she underwent retroperitoneoscopic right adrenalectomy with preservation of approximately 20% of adrenal tissue. Histology confirmed pheochromocytoma. Postoperatively, the patient’s blood pressure and metanephrine levels normalized (metanephrine level of 0.19 nmol/L and normetanephrine level of 0.59 nmol/L), and urinary frequency significantly improved. It is possible that pheochromocytoma had contributed to the urinary frequency in this patient.^3^ This case, thus, highlights that widespread FDG-avid brown fat activation is a powerful radiologic clue to the diagnosis of pheochromocytoma.

## Disclosure

The authors have no conflicts of interest to disclose.
